# One-Pot Cu/SAPO-34 for Continuous Methane Selective Oxidation to Methanol

**DOI:** 10.3390/molecules29102273

**Published:** 2024-05-11

**Authors:** Lanlan Sun, Yu Wang, Xuesong Gu, Meng Zhao, Lijuan Yuan

**Affiliations:** 1 Department of Application Chemistry, College of Biotechnology and Food Science, Tianjin University of Commerce, Tianjin 300134, China; guxuesong@tjcu.edu.cn (X.G.); zhaozh@tjcu.edu.cn (M.Z.); 19565371051@163.com (L.Y.); 2 School of Materials Science and Engineering and National Institute for Advanced Materials, Nankai University, Tianjin 300350, China; 2120181008@mail.nankai.edu.cn

**Keywords:** Cu/SAPO-34, MtM, one-pot synthesis, reaction condition, physical-phase characterization

## Abstract

Cu/SAPO-34 synthesized via a one-pot method with relatively low silicon content and copper loading at around 2 wt.% facilitated continuous oxidation of methane to methanol with a methanol space time yield of 504 μmol_CH3OH_/g_cat_/h. Remarkably, the methanol yield exceeded 1800 mmol_CH3OH_/mol_Cu_/h at 623 K. Typically, the presence of trace oxygen in the system was the key to maintaining the high selectivity to methanol. Characterization results from a series of techniques, including XRD, SEM, TEM, H_2_-TPR, NH_3_-TPD, UV-vis, and FTIR, indicated that Cu^2+^ existed in the position where it moves from hexagonal rings to elliptical cages as the active center.

## 1. Introduction

Efficient methane selective oxidation to methanol (MtM) has garnered significant attention in recent years due to its potential as a clean and efficient energy conversion process. Despite decades of research, developing suitable catalysts for this process in mild conditions faces multiple challenges, primarily due to the high reactivity of primary oxidation products [1,2].

Among the catalysts proposed for methane selective oxidation to methanol, zeolite catalysts have attracted attention due to their strong local electric fields, uniform channel or cage structures, and their capacity to form specific active metal species. These catalysts facilitate the selective absorption of reactants and products, resembling a structure similar to methane monooxygenase in nature [3,4,5,6,7]. Notably, the small-pore Cu-SSZ-13 zeolite catalyst, prepared via ion exchange, has demonstrated the ability to perform continuous methane selective oxidation to methanol, achieving a methanol space time yield (STY) of 542 mmol_CH3OH_/mol_Cu_/h at 573 K [8]. Through species optimization, its activity has been increased to 2678 mmol_CH3OH_/mol_Cu_/h [9]. Its active center is believed to be mononuclear Cu-OH or binuclear Cu-O-Cu [8,10,11]. In the continuous reaction, compared with common catalysts for MtM, the methanol production rate of Cu-SSZ-13 (based on per gram) exceeds that of Cu-MOR by more than two-fold [7,11,12] and surpasses Cu-ZSM-5 by more than four-fold [13,14]. The reason why small-pore zeolite achieves high selectivity in methane oxidation to methanol is its ability to stabilize active Cu species.

While Cu/SSZ-13 catalysts exhibit advantages in terms of hydrothermal stability and activity, a considerable portion of SSZ-13 zeolite syntheses still utilize traditional hydrothermal processes. These processes require the use of organic structure-directing agents, such as N,N,N-trimethyl-1-adamantammonium, and involve multiple steps of solid separation and calcination to obtain the final zeolite product. Consequently, the preparation process is intricate and costly [15]. Given these challenges, researchers have increasingly sought simplified and cost-effective methods for producing high-performance Cu-SSZ-13 zeolite catalysts. This exploration also takes into account SAPO-34 molecular sieves, which possess a CHA structure analogous to SSZ-13. However, there is scarce research on SAPO-34, a small-pore zeolite with the same structure as SSZ-13.

To date, there are several methods for loading copper onto SAPO-34 zeolites: (1) liquid-phase ion exchange, in which the active metal cationic components are exchanged with the ion exchange sites on the zeolite surface, thereby loading the active sites onto the corresponding carrier; (2) solid ion exchange, using a ball mill to physically and mechanically mix CuO with the SAPO-34 zeolite, followed by calcination at a certain temperature; and (3) chemical vapor deposition, in which metal salts are sublimated in a certain way, allowing them to enter the catalyst in a gaseous state, followed by calcination. Relevant studies have shown that effectively loading copper onto SAPO-34 zeolites via liquid-phase ion exchange is challenging, while the zeolite structure prepared by solid ion exchange is stable, but the zeolite skeleton can easily collapse during high-temperature roasting [16]. Additionally, Cu/SAPO-34 zeolites prepared by vapor deposition exhibit poor catalytic activity generally. Recently, a one-pot method for synthesizing Cu/SAPO-34 zeolite catalysts has gained attention [17]. This method allows for precise control of copper loading in the zeolite framework, regulation of the copper-to-silicon-aluminum ratio, and results in high catalytic activity and hydrothermal stability under harsh reaction conditions (high space velocity, high temperature, presence of water vapor) in the NH_3_-SCR reaction [18]. This is also crucial for studying methane selective oxidation to methanol. Furthermore, compared to the expensive templating agents required for synthesizing SSZ-13 zeolites, the synthesis of SAPO-34 is more economically efficient. In conclusion, the Cu/SAPO-34 zeolite catalyst synthesized using the one-pot method is well-suited for studying the selective oxidation of methane to methanol.

Herein, we addressed the challenge of methane selective oxidation to methanol, synthesized a series of Cu/SAPO-34 zeolites catalysts with different metal load contents using the one-pot method, optimized the catalysts by adjusting the metal loading, silicon content, crystallization time, and other synthesis conditions, and explored reaction conditions to obtain the best methanol yield. Subsequently, structural information of Cu/SAPO-34 was obtained through various characterization techniques to explore structure–activity relationship in the methane selective oxidation to methanol reaction.

## 2. Catalytic Performance in MtM

Liquid-phase ion exchange is a widely employed method for synthesizing Cu/SAPO-34 catalysts. However, this approach inevitably leads to the coexistence of preferred isolated Cu ions and undesired CuO, resulting in an uneven distribution of Cu within individual SAPO-34 particles. This complexity complicates the tracking and quantification of copper conversion under various hydrothermal treatments. To address the challenges arising from the complexity of the catalyst, a one-step process was employed to prepare a series of copper-modified SAPO-34 zeolite catalysts with varying metal load contents. As a comparison, a series of Cu-SAPO-34 was prepared by an ion exchange method by adjusting the concentration of the loaded metal salt (See Supplementary Materials for details).

**Figure 1:**
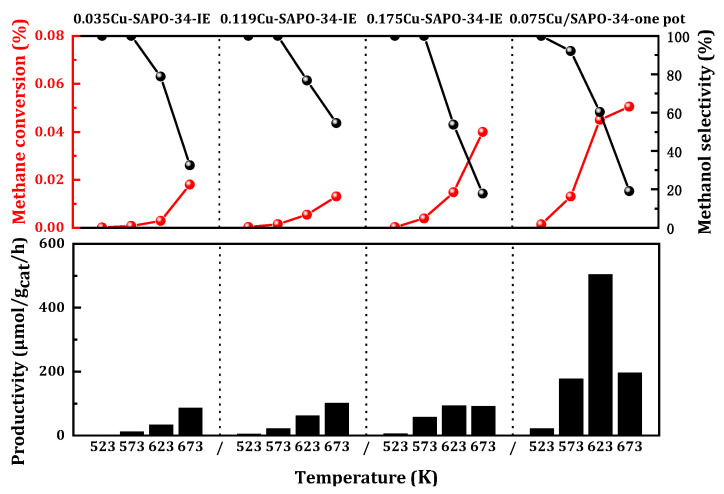
Catalytic performance of MtM conversion over the catalysts using the different synthetic methods. Reaction conditions: 0.1 g catalyst, total flowrate = 60 mL/min; 98% CH_4_, 2% H_2_O, 400 ppm O_2_.

The methane selective oxidation reaction took place within the temperature range of 523 to 673 K at atmospheric pressure. Both the catalysts prepared by the one-step and ion exchange methods exhibited catalytic activity (Figure 1). However, the catalysts synthesized using the one-step method demonstrate significant advantages. At 623 K, the yield can reach 504 μmol_CH3OH_/g_cat_/h, which was five times higher than that achieved using the ion exchange method (100 μmol_CH3OH_/g_cat_/h). The methane conversion rate was 0.045%, with a corresponding selectivity maintained at 65%. Consequently, the copper-modified Cu/SAPO-34 zeolite catalyst prepared using the one-step method held a distinct advantage for selective oxidation in methanol production.

**Figure 2:**
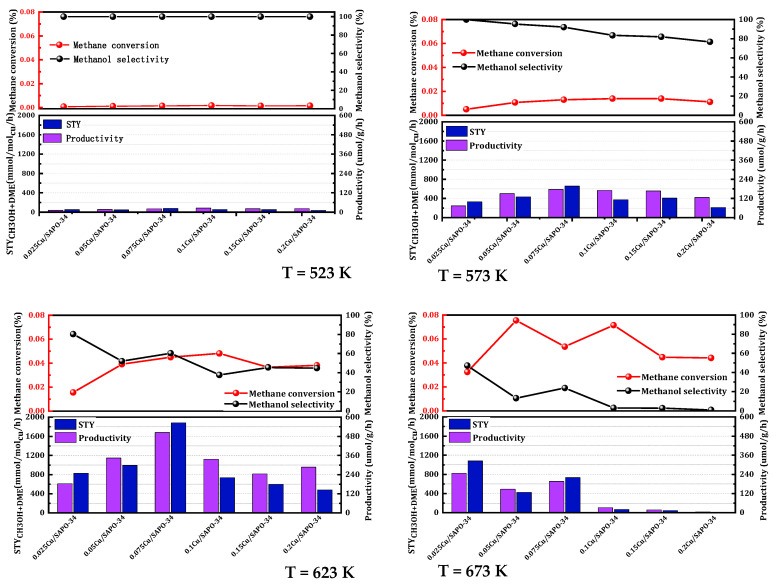
Catalytic performance of MtM conversion over the catalysts with various copper content. Reaction conditions: 0.1 g catalyst, total flowrate = 60 mL/min; 98% CH_4_, 2% H_2_O, 400 ppm O_2_.

It is well-established that the metal loading of a catalyst significantly influences its catalytic activity. To ascertain the optimal metal feeding proportion, the impact of copper loading on the methane oxidation activity of Cu/SAPO-34 was investigated and the copper loading using the aforementioned one-step synthesis method was optimized. As shown in Figure 2, the catalytic activity demonstrated a non-linear trend with the increase in copper load. The 0.075 Cu/SAPO-34 zeolite catalyst, with an actual copper load of 1.7%, exhibited the highest catalytic activity. When the copper load was below 1.7%, the activity gradually rose as the copper content increased. However, when the copper load exceeded 1.7%, methanol selectivity dropped to less than 50%, accompanied by a substantial generation of COₓ. Experimentally, deactivation occurred rapidly under high-temperature conditions with a high copper content. This could be attributed to the excessive occurrence of metal active sites leading to peroxidation reactions, methanol reforming, and oxidation reactions, ultimately forming carbon dioxide and reducing methanol selectivity. The 0.075 Cu/SAPO-34 catalyst demonstrated the most favorable copper content. This phenomenon also occurred in the NH_3_-SCR reaction, where Cu-SAPO34, an excellent catalyst, produced more active sites with approximately 2 wt% copper load, while a higher copper content would lead to increased oxides and reduced reaction conversion rates [19]. Additionally, Xue et al. [20] demonstrated that a high copper content affected the catalyst’s crystallinity and decreased active sites. Moreover, copper metal is prone to sintering and agglomeration under prolonged high-temperature reaction conditions, which also limits the activity of zeolite catalysts with higher copper content.

**Figure 3:**
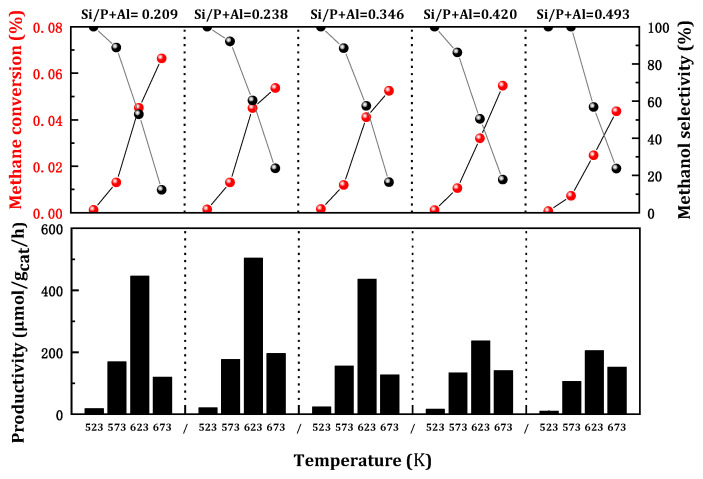
Catalytic performance of MtM conversion over the catalysts with different silicon content. Reaction conditions: 0.1 g catalyst, total flowrate = 60 mL/min; 98% CH_4_, 2% H_2_O, 400 ppm O_2_.

The impact of silicon content on the methane oxidation to methanol reaction is notably intriguing due to its influence on the acidity of SAPO-34 and the copper loading on the zeolite. It is widely recognized that the formation of Brønsted acid sites on SAPO-34 can be attributed to the binding of Si atoms to the neutral AlPO skeleton. The acidity intensity of the Si environment was observed to follow the sequence Si(1Al) > Si(2Al) > Si(3Al) > Si(4Al), representing the primary change in the Brønsted acid intensity of the Cu/SAPO-34 framework. Nevertheless, it has been demonstrated that the synthesized Cu/SAPO-34 exhibits low Si content and abundant Si(4Al) species, which can confer favorable catalytic activity and resistance to hydration [21]. To investigate the effect of Si content on activity, Cu/SAPO-34 catalysts with varying silicon content were prepared and their methane oxidation activity was evaluated subsequently (Figure 3). The methanol yield increased with silicon content, while methane conversion tended to decrease, accompanied by a slight rise in methanol selectivity. Optimal catalytic activity was observed at a relatively low Si content of Si/(P + Al) at 0.238. The results revealed that silicon content can impact the number of active sites in the reaction, but a high silicon loading was not conducive to the reaction. That is, the number of acid sites determined by the silicon content will also exert a certain influence on the activity.

**Figure 4:**
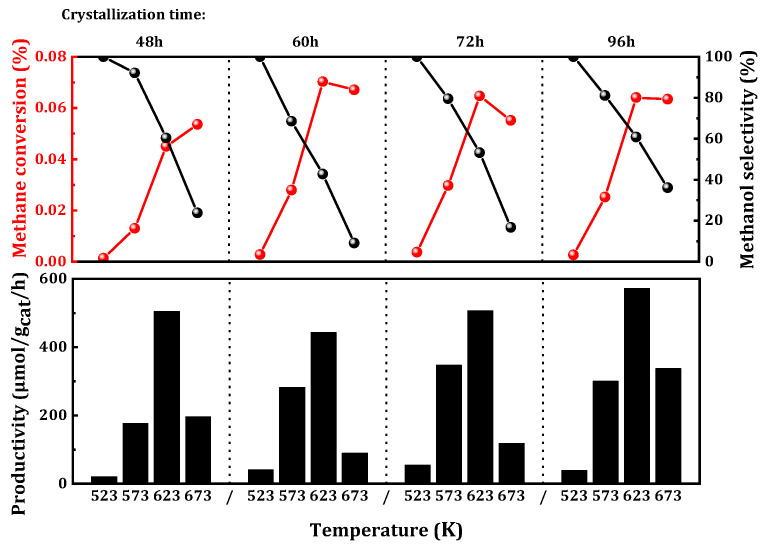
Catalytic performance of MtM conversion over Cu/SAPO-34 crystallized for different time. Reaction conditions: 0.1 g catalyst, total flowrate = 60 mL/min; 98% CH_4_, 2% H_2_O, 400 ppm O_2_.

Subsequently, the influence of crystallization time during zeolite synthesis on catalytic activity was investigated. Generally, longer crystallization times in the synthesis process lead to higher catalyst crystallinity, more regular morphology, and fewer defects. To explore the impact of temporal factors during crystallization on catalytic activity, the synthesis time of the catalyst was varied. The results of MtM are depicted in Figure 4. A crystallization time of 72 h demonstrated optimal catalytic performance at a lower temperature of 523 K. At 573 K, the catalyst with a crystallization time of 72 h maintained good catalytic activity (347 μmol_CH3OH_/g_cat_/h), with methane conversion reaching 0.03%, corresponding to a methanol selectivity of 79%. However, at relatively higher temperatures in the range of 623 to 673 K, the sample with a crystallization time of 96 h exhibited favorable catalytic effects (572 μmol_CH3OH_/g_cat_/h). This suggested that a shorter crystallization time was more favorable for the selective oxidation of methane at relatively low temperatures, while an increase in crystallization time at higher temperatures was beneficial to methane conversion.

**Figure 5:**
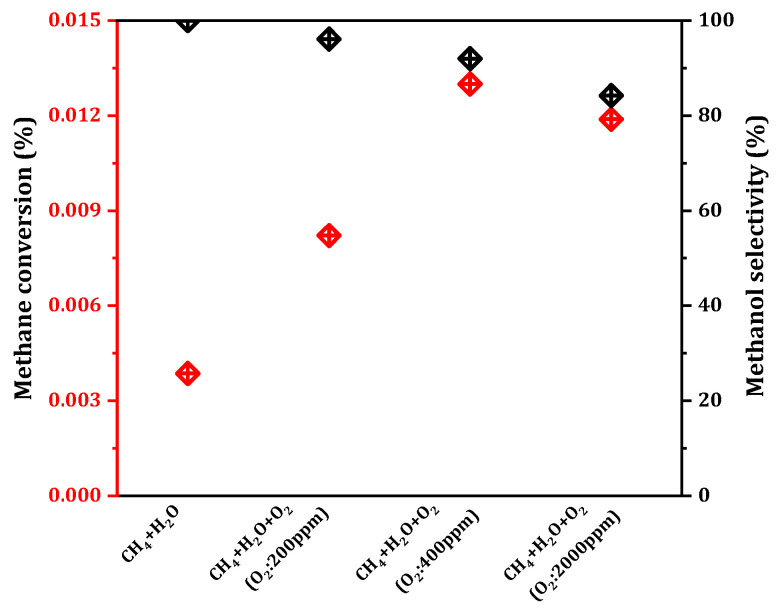
Catalytic performance (Methane conversion (red diamond) and Methanol selectivity (black diamond)) of MtM conversion over 0.075Cu/SAPO-34 under various oxygen content. Reaction conditions: 0.1 g catalyst, total flowrate = 60 mL/min; T = 573 K.

By screening the above preparation conditions, we prepared the 0.075Cu/SAPO-34 catalyst with high MtM activity, and then explored the reaction conditions in order to improve the methanol yield. The role of oxygen in the methane oxidation reaction has been a subject of debate here. While some studies have demonstrated that the oxidation role is primarily played by H_2_O in the methane oxidation [7], others have argued that O_2_ also holds significant importance in the oxidation process [22]. In light of this, the impact of O_2_ on the reaction was investigated by varying the concentration of O_2_ in the reaction system (from 0 to 2000 ppm). According to the results shown in Figure 5, in a trace amount of oxygen atmosphere (≤400 ppm), the conversion rate of methane increased as the oxygen concentration rose, and the methanol selectivity decreased slightly, but was still above 80%. The highest methane conversion, reaching 0.013%, was achieved at 400 ppm. Higher oxygen concentrations (>400 ppm) led to decreased methanol selectivity, with excessive oxygen resulting in the over-oxidation of methanol to CO_2_.

**Figure 6:**
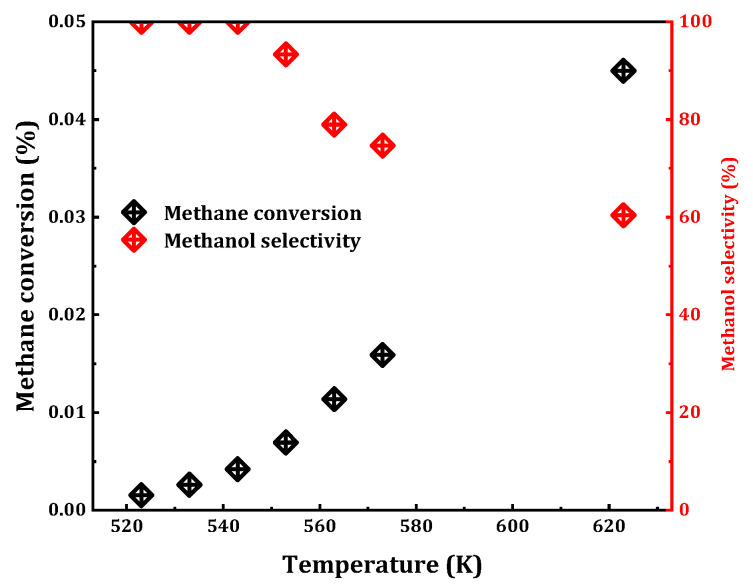
Catalytic performance of MtM conversion over 0.075Cu/SAPO-34 under different temperature. Reaction conditions: 0.1 g catalyst, total flowrate = 60 mL/min; 98% CH_4_, 2% H_2_O, 400 ppm O_2_.

For the vast majority of chemical reactions, the influence of temperature is of paramount importance. An increase in temperature often enhances reaction progression by providing the necessary thermal energy to overcome activation barriers. Building upon the aforementioned catalyst selection, the 0.075 Cu/SAPO-34 sample exhibiting relatively high catalytic activity was chosen for the reaction in the presence of trace oxygen (Figure 6). The results revealed that, with an increase in reaction temperature (523 K to 623 K), the methane conversion rate escalates from 0.002% to 0.045%, while the selectivity of methanol decreased from 100% to 61%. Below 543 K, methanol selectivity can be maintained at 100%, corresponding to a methane conversion rate of 0.004%. However, beyond this temperature, the appearance of the byproduct carbon dioxide led to reduced selectivity. At 573 K, the reaction exhibited favorable catalytic effects, maintaining a conversion rate of 0.016% and 75% selectivity. Consequently, for the MtM reaction on Cu/SAPO-34, 573 K was identified as the optimal reaction temperature.

**Figure 7:**
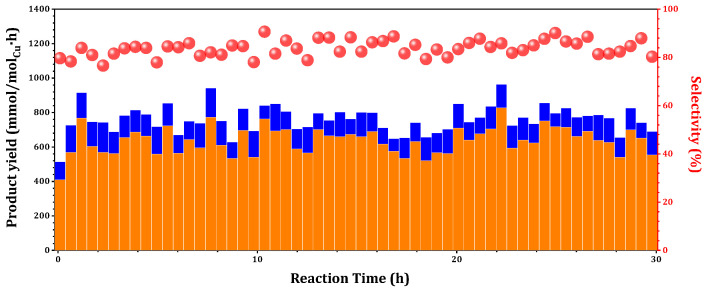
Time-on-stream behaviors of 0.075Cu/SAPO-34 in methane oxidation to methanol (orange column), carbon dioxide (blue column) and methanol selectivity (red circles). Reaction conditions: 0.1 g catalyst, total flowrate = 60 mL/min; 98% CH_4_, 2% H_2_O, 400 ppm O_2_.

The time-on-stream behaviors of 0.075Cu/SAPO-34 in MtM were further investigated. Experimentally, 0.075Cu/SAPO-34 demonstrated the production of methanol over a 30 h at 573K. The STY averaged 585 mmol_CH3OH_/mol_Cu_/h, with a conversion rate reaching 0.015% and corresponding to a remarkable 93% selectivity (Figure 7). According to previously reported methane conversion outcomes, when the methanol selectivity exceeds 90%, the methane conversion is limited to 0.1% [1]. Compared to the recently reported methanol yield (542 mmol_CH3OH_/molCu/h) [8], the one-pot Cu-SAPO-34 catalyst demonstrated promising potential and advantages.

## 3. Properties of MtM Catalysts

**Figure 8:**
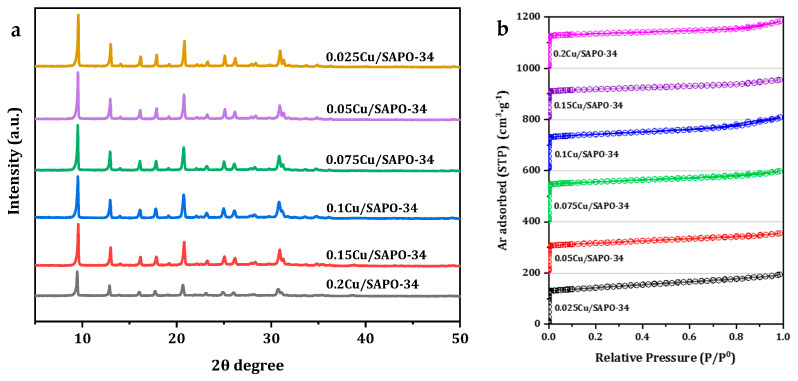
(**a**) XRD patterns of Cu-SAPO-34 via one-pot method employed; (**b**) Low-temperature Ar adsorption-desorption isotherms of Cu-SAPO-34 via one-pot method employed.

The structure of one-pot Cu/SAPO-34 catalysts was first investigated by means of XRD. As shown in Figure 8a, all the samples exhibited typical diffraction peaks characteristic of the CHA topology with similar intensities, and no characteristic diffraction peaks due to impurity could be observed, indicating that the sample had good crystallinity. Furthermore, the absence of diffraction peaks corresponding to copper oxide distinguished it from the typical diffraction peaks observed at 35.6° and 38.8° for Cu/SAPO-34 catalysts. This may be due to the ultrafine dispersion of copper species, or it may be because the copper content in the samples used in this work was below the detection limit of the instrument [23]. All the samples under study showed similar type I isotherms, characteristic of a zeolite microporous structure (Figure 8b). The textural properties of the zeolite samples under study are summarized in Table 1 for a direct comparison. All the samples exhibited similar BET surface areas of 356 to 480 m^2^/g and micropore volumes of 0.176 to 0.243 cm^3^/g, indicating the well-preserved CHA topology after the high-temperature calcination process. The actual metal loadings determined by ICP were slightly lower than the designated values due to the loss of Cu species during the filtration and calcination steps. Notably, the most active 0.075Cu/SAPO-34 zeolite catalyst sample maintained the maximum specific surface area and relatively higher micropore volume.

**Figure 9:**
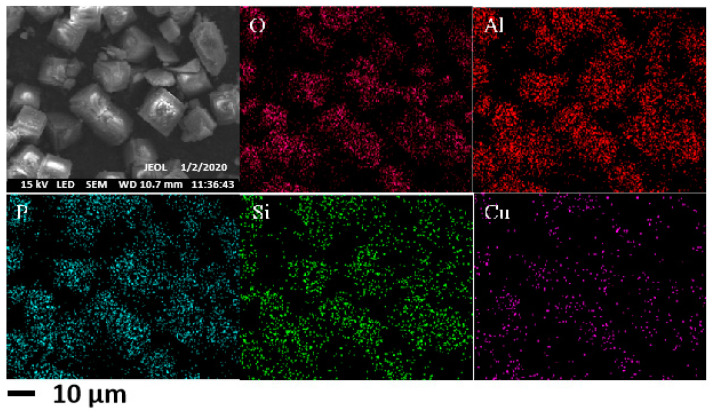
SEM images of 0.075Cu/SAPO-34 employed.

The surface morphology of copper-doped Cu/SAPO-34 catalyst was characterized using scanning electron microscopy (SEM), and the characterization results are shown in Figure 9. It can be directly observed from the figure that Cu/SAPO-34 exhibited a typical CHA cubic block structure, and the distribution of copper on the surface was relatively uniform. The SEM results were in basic agreement with the XRD results, indicating that the synthesized Cu/SAPO-34 was a typical silicon–phosphorus–aluminum zeolite catalyst.

**Figure 10:**
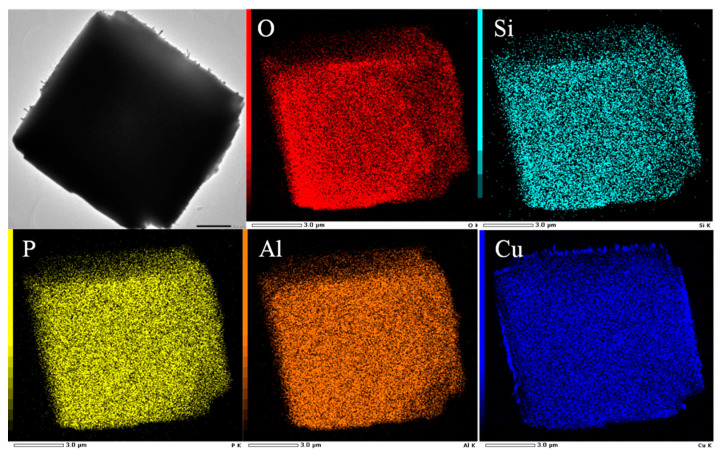
TEM images of 0.075Cu/SAPO-34 with corresponding element mapping analyses.

The distribution of Cu species on the SAPO-34 support could be directly observed by electron microscopy and the representative results are shown in Figure 10. As shown in the HR-TEM and HAADF-STEM images, after high-temperature calcination, Cu/SAPO-34 maintained a good particle morphology, with individual particle sizes remaining at around 11 μm. Meanwhile, metal agglomerates can be clearly observed on the surface of the samples in the selected-area elemental mapping analysis results, possibly due to the confinement effect of the zeolite pores on the metal species. Additionally, the combination of metal with water led to the formation of CuO_x_ particles loaded on the surface of the catalyst samples.

The redox properties of Cu species on the SAPO-34 support were investigated by means of H_2_-TPR. Based on the existing research results, the reduction peaks below 853 K were attributed to the reduction of surface Cu_x_O_y_ clusters on the zeolite framework to Cu^0^, while the reduction peaks at 563–583 K were attributed to the reduction of isolated Cu^2+^ ions to Cu^+^. In the high-temperature range of 673–1173 K, there are generally two hydrogen consumption peaks. The former occurs at 623–823 K when Cu^+^ is reduced to Cu^0^, where Cu^+^ mainly came from the reduction of isolated Cu^2+^ and the initially present Cu^+^ at the cation exchange sites of SAPO-34; the latter occurs at very high temperatures (973–1173 K) due to the reduction of framework Cu^+^ to Cu^0^, which can be described by the following simplified [24]:

Cu^2+^ + 1/2H_2_ = Cu^+^ + H^+^ or [Cu(OH)]^+^ + 1/2H_2_ = Cu^+^ + H_2_O (1)

CuO + H_2_ = Cu^0^ + H_2_O (2)

Cu^+^ + 1/2H_2_ = Cu^0^ + H^+^ (3)

**Figure 11:**
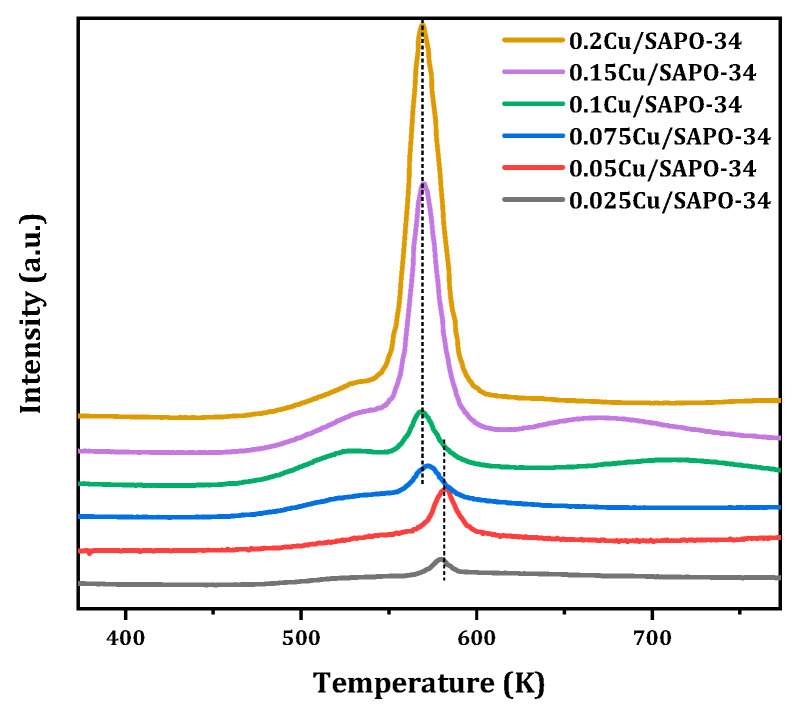
H_2_-TPR profile of Cu/SAPO-34 employed.

The results of Cu-SAPO-34 in this study are shown in Figure 11. When the copper content was low, the reduction peak of Cu_x_O_y_ clusters near 523 K increased with the increase of copper loading content, reaching a maximum value for 0.1Cu-SAPO-34. The reduction peak of isolated Cu^2+^ ions near 573 K also showed a significant variation pattern, and when the copper content is relatively low, this reduction peak appears at higher temperatures, whereas at higher copper contents, the reduction peak of Cu^2+^ ions gradually shifted towards lower temperatures. The reduction peaks above 673 K mainly came from the reduction of isolated Cu^2+^ to Cu^+^, mainly located in the region with higher copper loading. Currently, there is no Cu^+^ reduction peak on the framework above 973 K. It can be seen that copper species in Cu/SAPO-34 zeolite catalyst mainly exist in the form of isolated Cu^2+^ or partial Cu_x_O_y_, and no Cu^+^ entered the zeolite framework. In addition, the reduction temperatures of Cu^2+^ ions at different sites in the Cu/SAPO-34 structure were not the same. The reduction peak of Cu^2+^ ions near 563 K was generally attributed to the reduction of copper ions moving from hexagonal rings to elliptical cages; the Cu^2+^ located at the center of hexagonal prisms, i.e., double hexagonal rings, is generally difficult to reduce due to its higher coordination number and has a higher reduction temperature (583 K); in contrast, Cu^2+^ located at the eight-membered ring window was easier to reduce compared to other sites, requiring only a reduction temperature of 563 K. Therefore, it seems that, for Cu/SAPO-34 zeolite catalysts, Cu^2+^ was more likely to exist in the position where it moved from hexagonal rings to elliptical cages.

**Figure 12:**
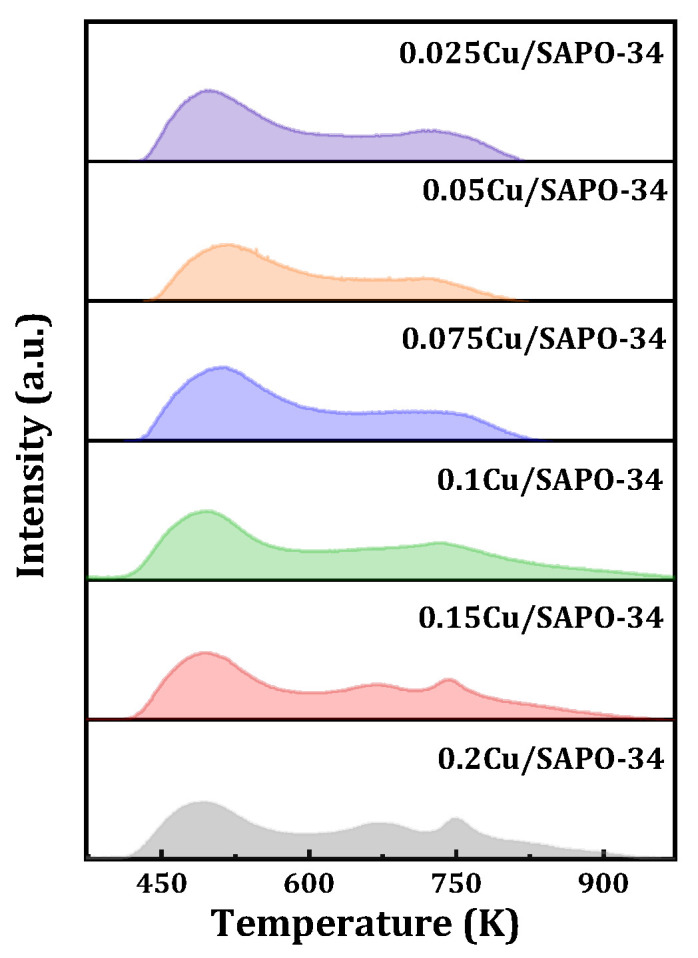
NH_3_-TPD profile of Cu/SAPO-34 employed.

The acidity of the samples was characterized by means of NH_3_-TPD, and the results are shown in Figure 12. For samples with lower copper content, two NH_3_ desorption peaks were observed. The low-temperature peak near 500 K is attributed to weak acid adsorption, generally associated with the desorption of physically adsorbed NH_3_ and NH_3_ adsorbed on weak Lewis acid sites (surface hydroxyl groups Si-OH, P-OH, Al-OH). The high-temperature peak near 743 K is attributed to strong acid adsorption, typically associated with the desorption of NH_3_ adsorbed on strong Brønsted acid sites and Lewis acid sites (Si-O(H)-Al and isolated Cu^2+^ ions). Furthermore, for samples with higher copper content, three NH_3_ desorption peaks were observed. These peaks were located near 493 K for weak acid desorption, around 670 K for moderate strong acid desorption, generally associated with the desorption of NH_3_ adsorbed on moderate Brønsted acid sites (Si-O(H)-Al), and near 743 K for moderate strong acid desorption [25]. In summary, it was found that, with the increase of loaded metal content, the strength of moderate strong acid increases while the strength of strong acid decreases. This may be mainly due to the introduction of excess copper in the one-step method, which affected the Cu^2+^ content and its interaction with the zeolite framework, especially the potential Cu-OH groups or interactions between Cu and acidic centers, and further acidity, leading to significant changes in the acidity environment of the framework.

**Figure 13:**
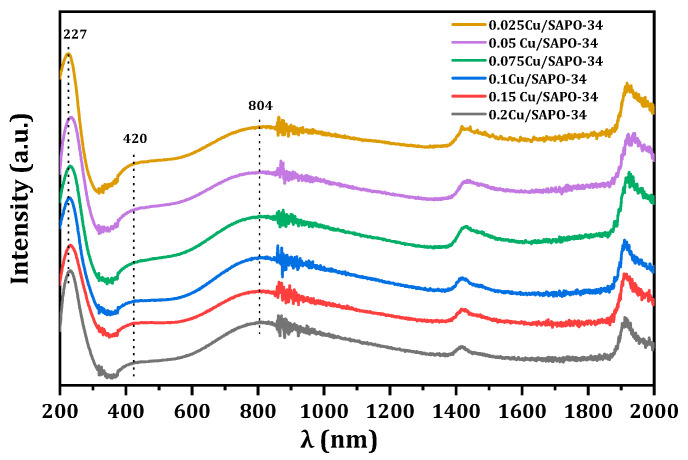
UV-Vis-NIR spectra of Cu/SAPO-34 under ambient conditions.

To understand the coordination and dispersion of Cu species in Cu/SAPO-34 catalysts with different copper contents, and to determine the influence of zeolite topology and composition on metal sites, the UV-Visible absorption spectra of the samples were studied. The results, as shown in Figure 13, revealed three main absorption bands for all samples. The UV absorption peak at around 240 nm was attributed to the charge transfer transition from ligands to isolated metal cations (O^2−^→Cu^2+^) [26,27,28]. Due to the strong water adsorption capacity of Cu/SAPO-34 zeolites, the UV absorption peak at 420 nm was assigned to hexa-coordinated Cu(H_2_O)_6_, Cu-O-Cu, and O-Cu-O species formed by metal and water [29,30,31]. Additionally, the UV absorption at 600–800 nm was attributed to the electron d-d transition of Cu^2+^ in a distorted octahedral coordination environment surrounded by oxygen [32]. Overall, the spectra indicated that, under these conditions, Cu primarily existed in the form of Cu^2+^ in the newly prepared catalysts.

**Figure 14:**
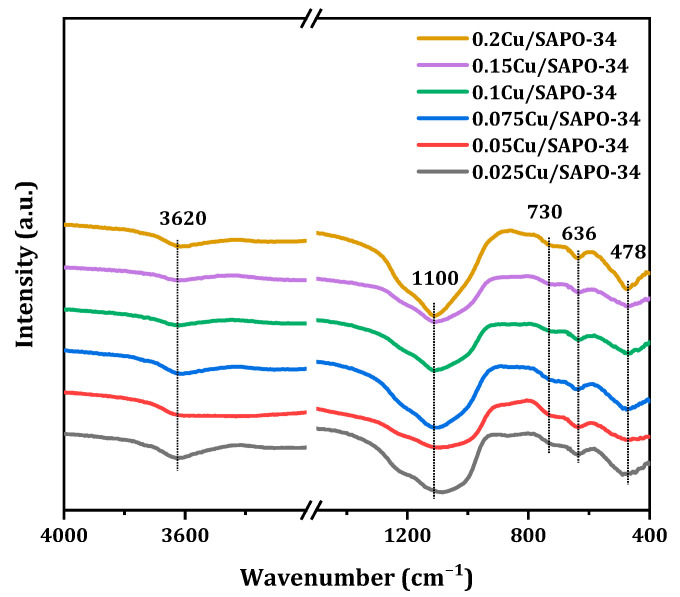
FTIR spectra of Cu/SAPO-34 employed.

The backbone structure of the synthetic molecular sieve was characterized by FT-IR (Figure 14). In order to determine whether copper was involved in the composition of the skeleton, the Cu/SAPO-34 zeolites were characterized and analyzed infrared. Compared with the infrared spectrum analysis of the SAPO-34 zeolite, the Cu/SAPO-34 zeolite has a similar skeleton vibration absorption band [33]. It follows that the vibration peak near 478 cm^−1^ belonged to T-O bending vibration of silicon oxygen tetrahedra; the 636 cm^−1^ peak belonged to the vibration of double six element rings; the peak near 730 cm^−1^ belonged to O-P-O or O-O-Al-O; peaks near 1100 cm^−1^ belonged to the asymmetric stretching vibration of O-P-O; and the 3620 cm^−1^ belonged to the bridge hydroxyl vibration of Al-O(H)-Si. According to the information measured from the skeleton infrared, any absorption corresponding to the vibration of Cu with the oxygen in the framework was not detected. Therefore, the Cu/SAPO-34 zeolite catalysts with different copper loads were not involved in the formation of the skeleton but may fell on the surface or pore of the catalyst.

**Figure 15:**
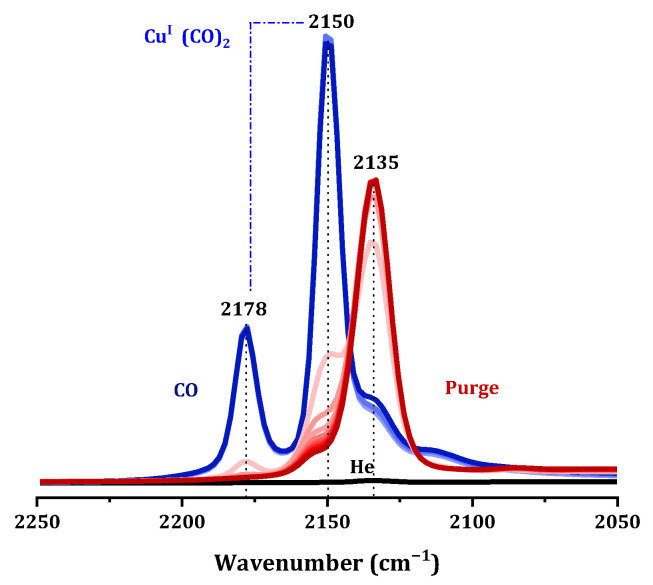
The CO adsorption/desorption DRIFTS onto 0.075Cu/SAPO-34 that was CO atmosphere (blue), and CO purge process (red from light to deep).

We know that copper species in catalysts can exist in the forms of Cu^+^ and Cu^2+^. To differentiate between various copper sites, FTIR spectroscopy of CO adsorption, the most common probe molecules for studying Cu^+^ and Cu^2+^ species, was used [7]. At room temperature, Cu^2+^ ions cannot adsorb CO to form stable compounds; when Cu^+^ was present in the sample, it can combine with CO to formed stable Cu^+^-CO carbonyl species, and an infrared absorption peak appeared in the wavenumber range of 2200–2080 cm^−1^, indicating chemisorption. In the infrared absorption spectrum in Figure 15, the 2150 cm^−1^ and 2179 cm^−1^ frequency vibrations were attributed to Cu^+^(CO)_2_ dicarbonyl species, indicating that the Cu species undergo self-reduction in a CO atmosphere [34]. As CO was purged, the infrared absorption of Cu-SAPO-34 gradually shifts towards a frequency of 2135 cm^−1^. This absorption at that position was attributed to the linearly adsorbed CO on the Cu^+^ surface, which may originate from the Cu^+^ present in the sample itself or from in situ reduction of Cu^+^ during the CO adsorption process (E^0^_Cu2+/Cu+_ = 0.153 eV) [35,36]. Taking into account the UV-vis and TPR data, it was indicated that on the fresh catalysts most of the copper was in the form of Cu^2+^, while IR study of adsorption of CO cannot unequivocally exclude the existence of Cu^+^. It was concluded that Cu^2+^ on Cu-SAPO-34 was very easily reduced into Cu^+^ by outgassing in the IR cell.

## 4. Materials and Methods

Zeolite host: SAPO-34 (Si/(P + Al) = 0.3), from Qilu Huaxin High-Tech Co. Ltd. (Shandong, China) and used as received.

Gases and chemicals: Methane (>99.995%), helium (>99.999%) and dioxygen (>99.995%) from Air Liquide Co. Ltd. (Tianjin, China), Copper sulfate pentahydrate (analytical reagent), i.e., CuSO_4_·5H_2_O, Copper actetate monohydrate (analytical reagent), i.e., Cu(CH_3_COO)_2_·H_2_O, Morpholine (99%), propan-2-ol (99%), i.e., C_3_H_8_O and Silica sol, i.e., SiO_2_, from Alfa Aesar Chemical Co. Ltd. (Shanghai, China); Phosphoric acid (85%), i.e., H_3_PO_4_, Tetraethylenepentamine from Macklin Chemical Co. Ltd. (Shanghai, China).

### 4.1. Preparation of Cu-Zeolite via One-Pot Method

A one-pot method was used to synthesize Cu/SAPO-34 zeolites with varying copper loadings. The catalyst feed ratio is: Al_2_O_3_: 0.9P_2_O_5_: 0.7SiO_2_: xCuO: 2 morpholine: yTEPA: mH_2_O, where x = 0.025–0.2, y = 0.025–0.2, and m = 30–40.

Step 1: The phosphorus source is mixed in water and stirred for five minutes. Subsequently, the aluminum source is added, and the mixture is stirred at room temperature for 1.5 h, resulting in the formation of the initial mixed solution.

Step 2: CuSO_4_·5H_2_O is mixed in deionized water. After complete dissolution, tetraethylenepentamine is added, and the solution is stirred for 30 min to create a copper-TEPA amine complex aqueous solution. The main template agent, silicon source, and each component are sequentially added and stirred for 30 min to generate the second mixed solution.

Step 3: The second mixed solution is combined with the first mixed solution and stirred at room temperature for 6–12 h. The resulting mixture is then transferred into a stainless-steel reactor lined with polytetrafluoroethylene and crystallized at 573 K for 2–4 days. Following crystallization, the reactor is cooled to room temperature, and the crystallized mixture is washed with 500 mL of deionized water to achieve neutrality. Subsequently, it is dried overnight in a 353 K oven and calcined at 823 K, resulting in the formation of the Cu/SAPO-34 zeolite catalyst. The samples obtained are designated as 0.025Cu/SAPO-34, 0.05Cu/SAPO-34, 0.075Cu/SAPO-34, 0.1Cu/SAPO-34, 0.15Cu/SAPO-34, and 0.2Cu/SAPO-34, based on the molar ratio of CuO/Al_2_O₃ in the feed.

For the preparation with varying crystallinity, the 0.075 Cu/SAPO-34 zeolite catalyst with a 1.7% copper metal load was selected to maintain a fixed copper content and a Si/(P + Al) ratio of 0.238 to uphold the optimal silicon content. The catalyst was crystallized into the reactor for 48, 60, 72, and 96 h.

The Cu-SAPO-34-IE zeolite catalyst was employed as a control, with copper contents of 3.5%, 11.9%, and 17.5% achieved through the ion exchange method. For clarity, the Cu-SAPO-34-IE catalyst with 3.5% copper content was selected for illustration. In this procedure, one gram of roasted SAPO-34 raw powder was introduced into a pre-prepared 0.01 mol/L copper acetate solution, followed by agitation at 303 K for 6 h. The resulting mixture underwent three washes with 500 mL of deionized water. Subsequently, the sample was dried in a 353 K oven overnight and roasted in air at 823 K for 6 h, leading to a recorded feed molar ratio of CuO/Al_2_O₃ of 0.156 for Cu-SAPO-34-IE.

### 4.2. Characterization of Cu-Zeolites

The X-ray diffraction (XRD) patterns of Cu-zeolite samples were recorded on a Bruker D8 diffractometer using Cu-Kα radiation (λ = 0.1541 nm) at a scanning rate of 6°/min in the region of 2θ = 5–50°.

The textual properties of Cu-zeolites were determined by argon adsorption-desorption isotherms at 87 K collected on a Quantachrome iQ-MP gas adsorption analyzer. The total surface areas were calculated via the Brunauer Emmett Teller (BET) equation and the micropore properties were determined to use the t-plot method.

Scanning electron microscopy (SEM) images of Cu-zeolite samples were obtained on a JSM-7500F electron microscope (JEOL, Tokyo, Japan).

Transmission electron microscopy (TEM) images of Cu-zeolite samples under study were acquired on a Tecnai G2 F20 electron microscope (FEI, Hillsboro, OR, United States). High angle annular dark filed scanning transmission electron microscopy (HAADF-STEM) images were acquired on a FEI Talos electron microscope. The element mapping analysis was performed under HAADF-STEM mode using a FEI built-in energy dispersive spectrum.

FTIR spectra of Cu-zeolite samples was collected on a Bruker Tensor 27 spectrometer in the diffuse reflectance mode with Harrick Praying Mantis setup and a liquid nitrogen cooled high sensitivity mercury-cadmium-telluride detector. The in situ diffuse reflectance FTIR spectroscopic analyses under different reaction conditions were performed in a Harrick CHC-CHA-3 chamber. The catalyst samples, after being ground, were loaded into an in situ pool, flattened, and subjected to pretreatment at 723 K in high-purity helium for 1 h before being reduced to the target temperature for testing. The instrument’s settings include a resolution of 4 cm⁻^1^, a scan number of 256, and a scan range of 600–4000 cm⁻^1^.

UV-vis-NIR spectra of Cu-zeolite samples were collected on a PerkinElmer Lambda 750 UV/VIS/NIR spectrometer in the diffuse reflectance mode with a Harrick Praying Mantis setup. The in situ UV-vis-NIR spectroscopic analyses under different reaction conditions were performed in an HVC-DRM-5 chamber. Sample processing followed the procedures outlined in the infrared spectrum. The UV absorption spectrum of the catalyst under various atmospheres was acquired against the background of the instrument’s configured whiteboard or powdered barium sulfate.

The hydrogen temperature-programmed reduction (H_2_-TPR) of Cu-zeolites was performed on a Quantachrome ChemBET 3000 chemisorption analyzer. In a typical experiment, Cu-zeolite sample of ca. 0.1 g was calcined in dry air at 673 K for 1 h and cooled to 323 K in flowing Ar. H_2_-TPR profile was recorded in flowing 10% H_2_/Ar at a heating rate of 10 K/min from 323 to 1023 K.

The experiment of NH_3_ temperature-programmed desorption (NH₃-TPD) was performed on a Quantachrome ChemBet 3000 chemisorption analyzer. In a typical experiment, the sample was saturated with 5% NH_3_/He at 323 K and then purged with He at the same temperature for 1 h to eliminate the physical absorbed species. The TPD profiles were recorded in flowing He at a heating rate of 10 K/min from 323 to 1073 K and the signals of desorption species were monitored by a Pfeiffer Omnistar GSD 320 mass spectrometer.

FTIR spectra of CO adsorption (CO-DFIRT) on selected samples was taken on a Bruker Tensor 27 spectrometer with 512 scans at a resolution of 2 cm^−1^. The sample was pretreated in He at 723 K for 1 h. After cooling to 298 K, the He stream was switched to 1% CO/He and time-dependent FTIR spectra of CO adsorption were sequentially recorded at 298 K.

### 4.3. Steady-State Methane Catalytic Oxidation to Methanol

**Figure 16:**
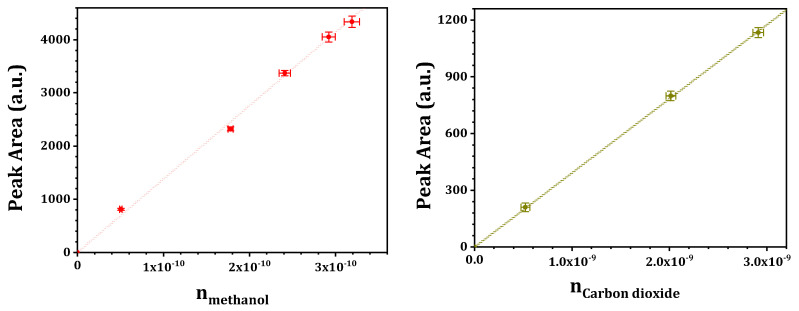
Typical calibration curves.

The selective catalytic oxidation of methane was performed on a fixed-bed micro-reactor at ambient pressure. Typically, catalyst sample of 0.1 g (sieve fraction 250–400 μm) was placed in the quartz reactor and the reactant gas mixture containing methane, water and dioxygen was fed to the quartz reactor at designated temperature. The total flow rate was controlled at 60 mL/min, corresponding to a gas hourly space velocity (GHSV) of 30,000/h. The reaction outlet was on-line analyzed by a gas chromatograph (SHIMADZU GC-2014, Kyoto City, Japan) equipped with a thermal conductivity detector (TCD, with one MS-13X packed column and two Porapak N packed columns) and a flame ionization detector (FID, with one Plot Q capillary column). Methane, carbon monoxide, carbon dioxide and dihydrogen were analyzed by TCD, while methane, C1 oxygenates and C2+ hydrocarbons were analyzed by FID (argon as an internal standard, methane as a link between TCD and FID). The concentration of methanol (also for carbon monoxide, carbon dioxide, etc.) was quantitatively determined by external standard method (see Figure 16 for typical calibration curves) and the yield of methanol ($Y_{\mathrm{CH}3\mathrm{OH}}$) was obtained via volume conversion. The methanol selectivity was determined by normalization: $S_{\mathrm{CH}3\mathrm{OH}}\left(\%\right)=\frac{{\left[{\mathrm{CH}}_{3}\mathrm{OH}\right]}_{\mathrm{outlet}}\;}{\sum \mathrm{Product}}*100\%$, (byproducts below the detect limitation of gas chromatograph were ignored and the possible coke deposition was fully excluded even after long-term running). The conversion of methane was calculated as: $C_{\mathrm{CH}4}\;\left(\%\right)=\frac{Y_{\mathrm{CH}3\mathrm{OH}}\;}{S_{\mathrm{CH}3\mathrm{OH}}}*100\%$.

## 5. Conclusions

The Cu/SAPO-34 was synthesized via a one-pot method and applied in methane catalytic oxidation in the presence of water and dioxygen under different conditions. The metal loading, silicon content, crystallization time, and other synthesis conditions were explored. The reaction temperature was raised from 473 to 723 K to promote methane conversion, and meanwhile, the concentration of dioxygen was regulated to minimize byproduct carbon dioxide production.

The 0.075 Cu/SAPO-34 zeolite catalyst, with an actual copper load of 1.7%, exhibited the highest catalytic activity, facilitating continuous oxidation of methane to methanol with a methanol space time yield of 504 μmol_CH3OH_/g_cat_/h. Remarkably, the methanol yield exceeded 1800 mmol_CH3OH/_mol_Cu_/h at 623 K. The presence of trace oxygen in the system (400 ppm) is the key to maintain the high selectivity toward desired product methanol.

Multiple means of characterization were confirmed that Cu^2+^ existed in the position where it moved from hexagonal rings to elliptical cages was the catalytically active center. This is highly consistent with the catalytic activity center for methane selective oxidation to methanol studied in Cu-SSZ-13 zeolite, but the one-pot synthesis of Cu/SAPO-34 avoids the use of expensive templates, which is more conducive to the industrial application of catalysts for methane selective oxidation to methanol.

## Figures and Tables

**Table 1:** Physicochemical properties of Cu/SAPO-34 with one-pot synthetic method.

Sample	S_BET_ (m^2^/g)	V_p_ (cm^3^/g)	Si/(P + Al)	Cu/Al	Composition of Cu/SAPO-34
0.025Cu/SAPO-34	480	0.243	0.135	0.017	1Al_2_O_3_:0.9P_2_O_5_:0.7SiO_2_:0.025 CuO:2MOR:0.025TEPA:30H_2_O
0.05Cu/SAPO-34	472	0.236	0.191	0.054	1Al_2_O_3_:0.9P_2_O_5_:0.7SiO_2_:0.05 CuO:2MOR:0.05TEPA:30H_2_O
0.075Cu/SAPO-34	462	0.233	0.228	0.063	1Al_2_O_3_:0.9P_2_O_5_:0.7SiO_2_:0.075 CuO:2MOR:0.075TEPA:30H_2_O
0.1Cu/SAPO-34	466	0.215	0.322	0.140	1Al_2_O_3_:0.9P_2_O_5_:0.7SiO_2_:0.1 CuO:2MOR:0.1TEPA:30H_2_O
0.15Cu/SAPO-34	389	0.189	0.354	0.153	1Al_2_O_3_:0.9P_2_O_5_:0.7SiO_2_:0.15 CuO:2MOR:0.15TEPA:30H_2_O
0.2Cu/SAPO-34	356	0.176	0.406	0.188	1Al_2_O_3_:0.9P_2_O_5_:0.7SiO_2_:0.2 CuO:2MOR:0.2TEPA:30H_2_O

## Data Availability

The original contributions presented in the study are included in the article/Supplementary Material, further inquiries can be directed to the corresponding authors.

## Supplementary Materials

The following supporting information can be downloaded at: https://www.mdpi.com/article/10.3390/molecules29102273/s1. Figure S1. Typical calibration curves.
