# Supplementary material for: One-Pot Cu/SAPO-34 for Continuous Methane Selective Oxidation to Methanol
Source: Molecules. 2024 May 11;29(10):2273. doi: 10.3390/molecules29102273 (PMC11124382; doi:10.3390/molecules29102273)
Supplement: Supplementary file 1 [file molecules-29-02273-s001.zip › molecules-2990305-supplementary.pdf]

Materials and Methods

Zeolite host: SAPO-34 (Si/P+Al =0.3), from Shandong Qilu Huaxin High-Tech Co. Ltd. and used as received.

Gases and chemicals: Methane (>99.995%), helium (>99.999%) and dioxygen (>99.995%) from Air Liquide (China) Co. Ltd.; Copper actetate monohydrate (analytical reagent), i.e. $Cu(CH_3COO)_2{\cdot}H_2O$, Morpholine (99%), propan-2-ol (99%), i.e. C_3H_8O and Silica sol, i.e. SiO_2, from Alfa Aesar (China) Chemical Co. Ltd.; Phosphoric acid (85%), i.e. H_3PO_4, Tetraethylenepentamine from Macklin (China) Chemical Co. Ltd.;

Preparation of Cu-zeolite via one-pot method

A one-pot method was used to synthesize Cu/SAPO-34 zeolites with varying copper loadings. The catalyst feed ratio is: Al_2O_3: $0.9P_2O_5$: $0.7SiO_2$: xCuO: 2 morpholine: yTEPA: mH_2O, where x=0.025-0.2, y=0.025-0.2, and m=30-40.

Step 1: The phosphorus source is mixed in water and stirred for five minutes. Subsequently, the aluminum source is added, and the mixture is stirred at room temperature for 1.5h, resulting in the formation of the initial mixed solution.

Step 2: $CuSO_4{\cdot}5H_2O$ is mixed in deionized water. After complete dissolution, tetraethylenepentamine is added, and the solution is stirred for 30 minutes to create a copper-TEPA amine complex aqueous solution. The main template agent, silicon source, and each component are sequentially added and stirred for 30 minutes to generate the second mixed solution.

Step 3: The second mixed solution is combined with the first mixed solution and stirred at room temperature for 6-12 hours. The resulting mixture is then transferred into a stainless steel reactor lined with polytetrafluoroethylene and crystallized at 573 K for 2-4 days. Following crystallization, the reactor is cooled to room temperature, and the crystallized mixture is washed with 500 mL of deionized water to achieve neutrality. Subsequently, it is dried overnight in an 353 K oven and calcined at 823 K, resulting in the formation of the Cu/SAPO-34 zeolite catalyst. The samples obtained are designated as 0.025Cu/SAPO-34, 0.05Cu/SAPO-34, 0.075Cu/SAPO-34, 0.1Cu/SAPO-34, 0.15Cu/SAPO-34, and 0.2Cu/SAPO-34, based on the molar ratio of CuO/Al_2O_3 in the feed.

For the preparation with varying crystallinity, the 0.075 Cu/SAPO-34 zeolite catalyst with a 1.7% copper metal load was selected to maintain a fixed copper content and a Si/P+Al ratio of 0.238 to uphold the optimal silicon content. The catalyst was crystallized into the reactor for 48, 60, 72, and 96 hours.

The Cu-SAPO-34-IE zeolite catalyst was employed as a control, with copper contents of 3.5%, 11.9%, and 17.5% achieved through the ion exchange method. For clarity, the Cu-SAPO-34-IE catalyst with 3.5% copper content was selected for illustration. In this procedure, one gram of roasted SAPO-34 raw powder was introduced into a pre-prepared 0.01 mol/L copper acetate solution, followed by agitation at 303 K for 6 hours. The resulting mixture underwent three washes with 500 mL of deionized water. Subsequently, the sample was dried in an 353 K oven overnight and roasted in air at 823 K for 6 hours, leading to a recorded feed molar ratio of CuO/Al_2O_3 of 0.156 for Cu-SAPO-34-IE.

Characterization of Cu-zeolites

The X-ray diffraction (XRD) patterns of Cu-zeolite samples were recorded on a Bruker D8

diffractometer using Cu-Kα radiation (λ= 0.1541 nm) at a scanning rate of 6 °/min in the region of 2θ = 5-50°.

The textual properties of Cu-zeolites were determined by argon adsorption-desorption isotherms at 87 K collected on a Quantachrome iQ-MP gas adsorption analyzer. The total surface areas were calculated via the Brunauer Emmett Teller (BET) equation and the micropore properties were determined to use the t-plot method.

Scanning electron microscopy (SEM) images of Cu-zeolite samples were obtained on a JSM-7500F electron microscope.

Transmission electron microscopy (TEM) images of Cu-zeolite samples under study were acquired on a FEI Tecnai G2 F20 electron microscope. High angle annular dark filed scanning transmission electron microscopy (HAADF-STEM) images were acquired on a FEI Talos electron microscope. The element mapping analysis was performed under HAADF-STEM mode using a FEI built-in energy dispersive spectrum.

FTIR spectra of Cu-zeolite samples was collected on a Bruker Tensor 27 spectrometer in the diffuse reflectance mode with Harrick Praying Mantis setup and a liquid nitrogen cooled high sensitivity mercury-cadmium-telluride detector. The in situ diffuse reflectance FTIR spectroscopic analyses under different reaction conditions were performed in a Harrick CHC-CHA-3 chamber. The catalyst samples, after being ground, were loaded into an in situ pool, flattened, and subjected to pretreatment at 723 K in high-purity helium for 1 hour before being reduced to the target temperature for testing. The instrument's settings include a resolution of 4 cm^{-1}, a scan number of 256, and a scan range of 600-4000 cm^{-1}.

UV-vis-NIR spectra of Cu-zeolite samples were collected on a PerkinElmer Lambda 750 UV/VIS/NIR spectrometer in the diffuse reflectance mode with a Harrick Praying Mantis setup. The in situ UV-vis-NIR spectroscopic analyses under different reaction conditions were performed in a HVC-DRM-5 chamber. Sample processing followed the procedures outlined in the infrared spectrum. The UV absorption spectrum of the catalyst under various atmospheres was acquired against the background of the instrument's configured whiteboard or powdered barium sulfate.

The hydrogen temperature-programmed reduction (H_2-TPR) of Cu-zeolites was performed on a Quantachrome ChemBET 3000 chemisorption analyzer. In a typical experiment, Cu-zeolite sample of ca. 0.1 g was calcined in dry air at 673 K for 1 hour and cooled to 323 K in flowing Ar. H_2-TPR profile was recorded in flowing 10% H_2/Ar at a heating rate of 10 K/min from 323 to 1023 K.

The experiment of NH_3 temperature-programmed desorption (NH_3-TPD) was performed on a Quantachrome ChemBet 3000 chemisorption analyzer. In a typical experiment, the sample was saturated with 5% NH_3/He at 323 K and then purged with He at the same temperature for 1 hour to eliminate the physical absorbed species. The TPD profiles were recorded in flowing He at a heating rate of 10 K/min from 323 to 1073 K and the signals of desorption species were monitored by a Pfeiffer Omnistar GSD 320 mass spectrometer.

FTIR spectra of CO adsorption (CO-DFIRT) on selected samples was taken on a Bruker Tensor 27 spectrometer with 512 scans at a resolution of 2 cm^{-1}. The sample was pretreated in He at 723 K for 1 hour. After cooling to 298 K, the He stream was switched to 1% CO/He and time-dependent FTIR spectra of CO adsorption were sequentially recorded at 298 K.

Steady-state methane catalytic oxidation to methanol

The selective catalytic oxidation of methane was performed on a fixed-bed micro-reactor at ambient pressure. Typically, catalyst sample of 0.1 g (sieve fraction 250-400 μm) was placed in the quartz reactor and the reactant gas mixture containing methane, water and dioxygen was fed to the quartz reactor at designated temperature. The total flow rate was controlled at 60 mL/min, corresponding to a gas hourly space velocity (GHSV) of 30,000 /h. The reaction outlet was on-line analyzed by a gas chromatograph (SHIMADZU GC-2014) equipped with a thermal conductivity detector (TCD, with one MS-13X packed column and two Porapak N packed columns) and a flame ionization detector (FID, with one Plot Q capillary column). Methane, carbon monoxide, carbon dioxide and dihydrogen were analyzed by TCD, while methane, C1 oxygenates and C2+ hydrocarbons were analyzed by FID (argon as an internal standard, methane as a link between TCD and FID). The concentration of methanol (also for carbon monoxide, carbon dioxide, etc.) was quantitatively determined by external standard method (see Figure S1 for typical calibration curves) and the yield of methanol (Y_{CH3OH}) was obtained via volume conversion. The methanol selectivity was determined by normalization: $S_{\mathrm{CH3OH}}(\%) = \frac{[\mathrm{CH_3OH}]_{\mathrm{outlet}}}{\sum \mathrm{Product}} * 100\%$, (byproducts below the detect limitation of gas chromatograph were ignored and the possible coke deposition was fully excluded even after long-term running). The conversion of methane was calculated as: $C_{\mathrm{CH4}}\ (\%) = \frac{Y_{\mathrm{CH3OH}}}{S_{\mathrm{CH3OH}}} * 100\%$.

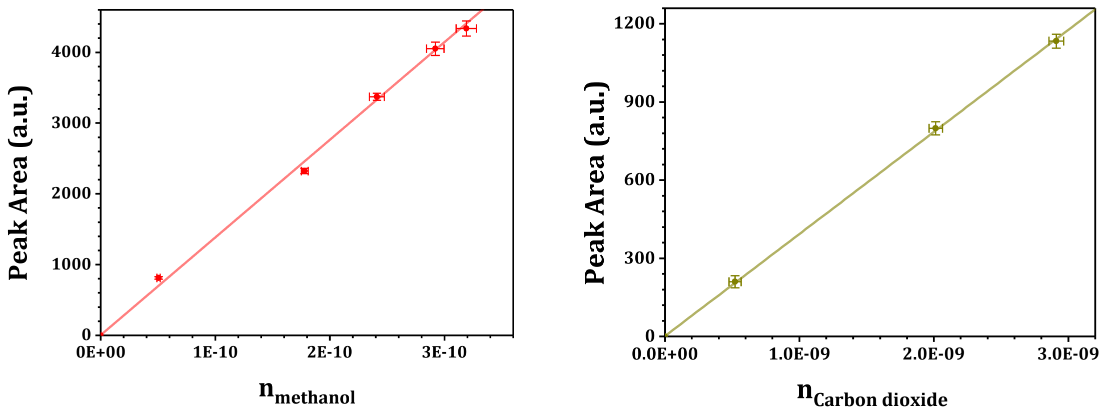

Figure S1. Typical calibration curves
